# Establishment and application of a dual chip digital PCR assay for detection of PDCoV and PEDV

**DOI:** 10.3389/fvets.2025.1655079

**Published:** 2025-09-15

**Authors:** Yue Zhang, Fangting Dong, Yuhang Zhang, Yutong Feng, Jinwang Hu, Yuhang Li, Lu Xia, Shaopo Zu, Hao Lu, Zhanyong Wei

**Affiliations:** ^1^College of Veterinary Medicine, Henan Agricultural University, Zhengzhou, China; ^2^Ministry of Education Key Laboratory for Animal Pathogens and Biosafety, Zhengzhou, China; ^3^Henan Province Key Laboratory of Animal Food Pathogens Surveillance, Zhengzhou, China; ^4^Molecule Biology Laboratory of Zhengzhou Normal University, Zhengzhou, China

**Keywords:** cdPCR, PDCoV, PEDV, dual assay, detection

## Abstract

**Introduction:**

Coinfection with porcine deltacoronavirus (PDCoV) and porcine epidemic diarrhea virus (PEDV) is a major cause of acute diarrhea in piglets, which poses a significant challenge to the swine industry. The early detection and control of these two viruses require highly sensitive diagnostic tools. We developed a novel chip digital PCR (cdPCR) assay that uses two probes for the simultaneous quantitative detection of both PDCoV and PEDV in clinical samples.

**Methods:**

In this study, the dual cdPCR reaction system, including the annealing temperature and primer–probe concentration ratio, was systematically optimized. Additionally, we validated the developed method for specificity, sensitivity, linearity, and repeatability. Finally, the method was applied to assess the biological samples with low viral loads.

**Results:**

The dual cdPCR assay demonstrated exceptional sensitivity, with limits of detection (LoD) of 1.83 ± 0.15 copies/μL for PDCoV and 0.99 ± 0.07 copies/μL for PEDV, high specificity (no cross-reactivity with TGEV, PSV, or PRV), outstanding linearity (*R^2^* = 0.9972 for PDCoV and *R^2^* = 0.9969 for PEDV) and reproducibility (intra- and inter-assay CV < 6%). Validation across 148 clinical samples indicates that our dual cdPCR is more sensitive than qPCR for detecting both single and mixed infections. Notably, this assay can effectively quantify PDCoV and PEDV in environmental aerosol samples.

**Discussion:**

Our results demonstrate that this dual cdPCR assay offers a highly sensitive, stable, and accurate platform for the simultaneous quantification of both PDCoV and PEDV. It represents a valuable tool for early disease monitoring (particularly in aerosol surveillance and mixed-infection scenarios with low viral loads), thereby supporting the effective prevention of porcine viral diarrhea and the sustainable growth of the swine industry.

## 1 Introduction

PDCoV and PEDV are typical enteric coronaviruses that cause acute infectious diseases in domestic pigs and wild boars—characterized by severe diarrhea, vomiting, weight loss, and high mortality (1). These two coronaviruses have significantly harmed the swine industry (2). PDCoV was first reported in Hong Kong, China, in 2012 through molecular surveillance studies, followed by the isolation of the first PDCoV OH-FD22 strain in the United States (3, 4). PDCoV gained widespread attention after a 2014 outbreak that spread rapidly to Canada, China, Vietnam, Laos, South Korea, and Mexico (5–8). PEDV was first detected in the United Kingdom and Belgium in the late 1970s and has since spread worldwide, causing substantial economic losses (9). Mixed infections with PDCoV and PEDV are common in swine farms. Among diarrheal pigs in China, coinfection with PDCoV and PEDV has reportedly reached as high as 60.4% in Henan Province and 55.9% in Sichuan Province (1, 10). PDCoV and PEDV coinfections were also prevalent in pig diarrhea samples collected in South Korea (11). As of yet, there are no effective vaccines or drugs available to treat and prevent PDCoV and PEDV infections, making it imperative to develop a rapid, sensitive, efficient, and accurate tool for detecting these viruses to mitigate widespread transmission.

PDCoV and PEDV are primarily transmitted through the fecal-oral route. However, some studies have highlighted the potential role of aerosol transmission (12, 13). Vitosh-Sillman et al. (13) reported that a PDCoV aerosol-infected group developed diarrhea, demonstrating that the virus can be aerosolized under conditions of confinement. Alonso et al. (14) investigated the spatial distributions of aerosolized PEDV and observed higher infection rates in proximity to positive locations, providing evidence for the long-distance airborne spread of PEDV (15). Subsequent research on the mechanisms of airborne PEDV transmission revealed that CD3^+^T cells carry the virus to the intestine through blood circulation after the nasal epithelium is infected (16). Given these viruses’ impact on pigs and the challenges associated with controlling airborne pathogens, monitoring aerosols for PDCoV and PEDV particles demands a sensitive and precise technical approach.

In nanofluidic chip digital PCR (cdPCR) assaying, samples are distributed to thousands of reaction wells for independent amplification. The average statistical probability of detecting positive fluorescence signals in each reaction unit is calculated using the Poisson distribution (17–19). A defining feature of cdPCR is its intrinsic capacity for absolute nucleic acid quantification without reliance on external calibration curves or reference genes. This methodology fundamentally eliminates amplification efficiency-associated variations, thereby enabling direct and highly reproducible quantification of targets (20). It can also detect viral emergence at extremely low concentrations, thereby facilitating early detection (21). In this study, we developed a dual cdPCR assay incorporating both the FAM and HEX channels. This enables the simultaneous detection of both PDCoV and PEDV in samples, including aerosolized and mixed-infection samples.

## 2 Materials and methods

### 2.1 Viruses, primers, and probes

The highly virulent PDCoV HNZK-02 strain (GenBank: MH708123.1) and the PEDV HN-2021 strain (GenBank: OR707084.1) were used in this study. The DNA/cDNA samples of transmissible gastroenteritis virus (TGEV), porcine sapelovirus (PSV), and porcine pseudorabies virus (PRV) were isolated in the Ministry of Education Key Laboratory for Animal Pathogens and Biosafety, and stored at −80 °C.

Primer Premier (version 5.0) was used to design primers and probes to target the N gene of PDCoV and the M gene of PEDV. The probes for PDCoV and PEDV were labeled with FAM and HEX at the 5′ end, respectively, as well as BHQ1 quenchers at all the 3′-ends. The primer and probe sequences are listed in Table 1.

**Table 1:** The primers and probes for detection of PDCoV and PEDV.

Primers and probes	Sequences (5′–3′)
PDCoV-N-Forward	CCCTTACCTTCTCGTACTCAATC
PDCoV-N-Reverse	GTTTGGTGGGTGGCTCATA
PDCoV-N-FAM	FAM-AAGGATGCGCTCAATACGGTCGTT-BHQ1
PEDV-M-Forward	TGGTCTTTCAATCCTGAAACAGA
PEDV-M-Reverse	GAGTGTTAGCGTTACACCAGTT
PEDV-M-HEX	HEX-AGGTCTGCATTCCAGTGCTTGGA-BHQ1

### 2.2 Clinical samples and nucleic acid extraction

Diarrhea samples were collected from piglets in Henan, Hebei, and Shanxi provinces between December 2020 and January 2024. The intestinal tissues were divided into 2 mL EP tubes, and the volume was adjusted to 1.5 mL using MEM. It was then ground repeatedly until a paste-like consistency was achieved, followed by three cycles of freeze-thawing. The samples were then centrifuged at 3,000 rpm for 20 min at a temperature of 4 °C. The supernatant was then filtered through a microporous filter with a pore size of 0.22 μm to yield the sample filtrate for RNA extraction. After lysing the samples with TRIzol, the total RNA was extracted using a Nucleic Acid Extraction & Purification Kit (Fastagen Biotech, Shanghai, China) and subsequently reverse transcribed into cDNA using the HiScript III 1st Strand cDNA Synthesis Kit (+gDNA wiper) (Vazyme, Nanjing, China).

### 2.3 Standard plasmid preparation

The N and M gene sequences of PDCoV and PEDV were amplified via PCR and subsequently cloned into the pMD18-T vector. The resulting plasmids were designated as pMD18-T-PDCoV-N and pMD18-T-PEDV-M, respectively. After the plasmids were extracted using a plasmid extraction kit, their concentrations were measured using a NanoDrop Vue Plus ultrafine spectrophotometer (Thermo Fisher Scientific, USA). The concentration of PDCoV plasmids was 3.36 × 10^9^ copies/μL, while that of PEDV plasmids was 2.12 × 10^9^ copies/μL. To create varying concentrations for the subsequent experiments, the corresponding copy number gradients were diluted within the ranges of 3.36 × 10^9^–3.36 × 10^0^ copies/μL for PDCoV, and 2.12 × 10^9^–2.12 × 10^0^ copies/μL for PEDV. The number of plasmid DNA copies was calculated using the following formula: amount (copies/μL) = [DNA concentration (ng/μL) × 10^−9^] / (plasmid lengths in base pairs × 660) × (6.02 × 10^23^).

### 2.4 Optimizations for the dual cdPCR assay

The experimental conditions, including primer and probe concentration ratios and annealing temperature, were systematically refined. Leth et al. (22) demonstrated that the detection performance was optimal when the concentrations of forward and reverse primers were equal. Following the previous research about primer-probe concentration ratios (23, 24), we configured the reaction system with the following combinations: 300 nM/200 nM, 600 nM/500 nM, 800 nM/1000 nM, 1,000 nM/1000 nM, 1,000 nM/1500 nM, and 500 nM/1000 nM. The reactions were optimized based on the primer annealing step within a temperature range of 52–60 °C.

First, the reaction system and amplification chip were prepared. The prepared nucleic acids were used as detection templates and mixed with the Digital PCR Universal Kit (Shanghai Little Turtle Technology Co.). Subsequently, the reaction system and oil-phase plate were placed into an automatic mixing system to prepare the chip. Next, the digital PCR automatic droplet preparation and amplification instruments were used to perform the reaction and amplification cycles. The PCR cycling conditions were set at 95 °C for 2 min, 45 cycles of denaturation at 95 °C for 15 s, and annealing/extension at 58 °C for 30 s. Finally, the chips were placed into a digital PCR data analyzer for fluorescence imaging to read the data acquired by the FAM and HEX dual signal channels. The samples’ positive and negative droplets were analyzed using the Biochip Data Analysis software. No template control (NTC) was used as the negative control.

### 2.5 Sensitivity analysis

Ten-fold serial dilutions of pMD18-T-PDCoV-N (at 3.36 × 10^9^–3.36 × 10^0^ copies/μL) and pMD18-T-PEDV-M (at 2.12 × 10^9^–2.12 × 10^0^ copies/μL) were used to evaluate the sensitivity of the dual cdPCR. The LoD was determined using GraphPad Prism version 7.4 (GraphPad Software, LA Jolla, CA, USA).

### 2.6 Repeatability

The pMD18-T-PDCoV-N standard positive plasmid (at concentrations of 3.36 × 10^3^ copies/μL, 3.36 × 10^2^ copies/μL, and 3.36 × 10^1^ copies/μL) and pMD18-T-PEDV-M (at concentrations of 2.12 × 10^3^ copies/μL, 2.12 × 10^2^ copies/μL, and 2.12 × 10^1^ copies/μL) were used as templates for cdPCR amplification, in order to evaluate the repeatability of our dual cdPCR method. All the reactions were repeated in triplicate within and between each group.

### 2.7 Aerosol sample detection

The study involved a group of nine five-day-old piglets with similar weights. A comprehensive panel of pathogen tests was conducted to ensure the subjects were PDCoV- and PEDV-negative. The piglets were then randomly assigned to three distinct groups: the PEDV-inoculated group, the PDCoV-inoculated group, and the negative control group. The two experimental groups were orally administered 10 mL of PDCoV strain (at a concentration of 2 × 10^6.0^ TCID_50_/mL) and 2 mL of PEDV strain (at a concentration of 1 × 10^5.0^ TCID_50_/mL), respectively. The control group was administered the same volume of Dulbecco’s Modified Eagle’s Medium (DMEM). Three piglets in the PEDV-inoculated group died less than 3 days after administration, whereas no deaths were observed in the PDCoV-inoculated group. An aerosol sampler (ASP-200p, Shenzhen Lemniscare Medical Technology Co., Ltd., China) was used to collect air samples (at horizontal distances of 0 m and 2 m and a sampling height of 0.6 m) from the positive isolation point at 24, 48, and 72 h following oral administration of the virus. Due to the deaths of the piglets in the PEDV-inoculated group, no air samples were collected at the 72-h mark. The RNA copy numbers of PDCoV and PEDV in the aerosols were then determined using an established digital PCR method.

The animal experiments were conducted in strict accordance with the Guidelines for Experimental Animals of the Ministry of Science and Technology (Beijing, China) and received approval from the Institutional Animal Care and Use Committee (IACUC) of Henan Agricultural University (approval number: HNND2022030811).

### 2.8 Statistical analysis

The statistical analyses, consistency estimation, and linear regression were performed using GraphPad Prism version 7.4 and SPSS version 26.0 (IBM, Armonk, NY, USA).

## 3 Results

### 3.1 Optimizing the dual cdPCR reaction system

**Figure 1:**
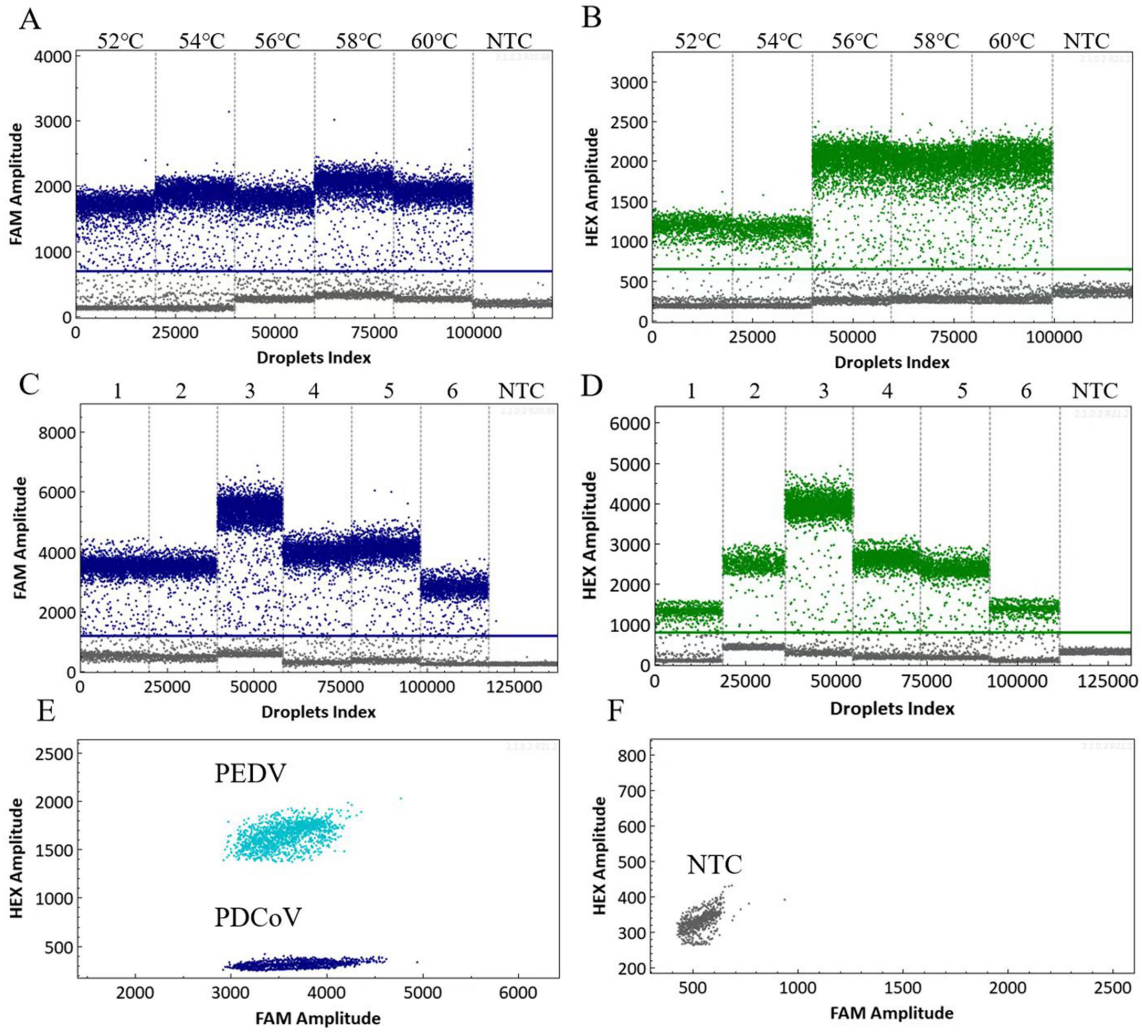
Optimized scatter plot of the annealing temperature for the dual cdPCR detection of PDCoV **(A)** and PEDV **(B)**. Determination of the optimal primer–probe concentration ratio for the dual cdPCR detection of PDCoV **(C)** and PEDV **(D)**. 1–6: 300 nM/200 nM; 600 nM/500 nM; 800 nM/1000 nM; 1,000 nM/1000 nM; 1,000 nM/1500 nM; and 500 nM/1000 nM. Two-dimensional dual-channel droplet generation diagram for PDCoV and PEDV **(E,F)**. NTC, No-template control.

To enhance the precise detection of PDCoV and PEDV, it is imperative to optimize the cdPCR detection procedure. The annealing temperature and primer–probe concentration ratios are important parameters within the reaction system that directly affect the droplets’ generation number and fluorescence intensity (25). The dual cdPCR amplification was performed by setting five layers at 52 °C, 54 °C, 56 °C, 58 °C, and 60 °C. Our results indicated that 58 °C was the optimal annealing temperature (Figures 1A,B). At this temperature, a noticeable separation was observed between the positive and negative droplets. The primer–probe concentration ratios for the reaction system configuration were set to 300 nM/200 nM, 600 nM/500 nM, 800 nM/1000 nM, 1,000 nM/1000 nM, 1,000 nM/1500 nM, and 500 nM/1000 nM. The optimal concentration ratio for both PDCoV and PEDV was 800 nM/1000 nM (Figures 1C,D). This ratio of reagents resulted in the largest difference in fluorescence amplitude between the positive and negative droplet clusters. The two-dimensional dual-channel droplet generation diagram showed that only the corresponding FAM and HEX signals for PDCoV and PEDV could be specifically detected, and that the two viruses’ fluorescence signals did not interfere with each other (Figures 1E,F).

### 3.2 Specificity and sensitivity analysis of the dual cdPCR assay

**Figure 2:**
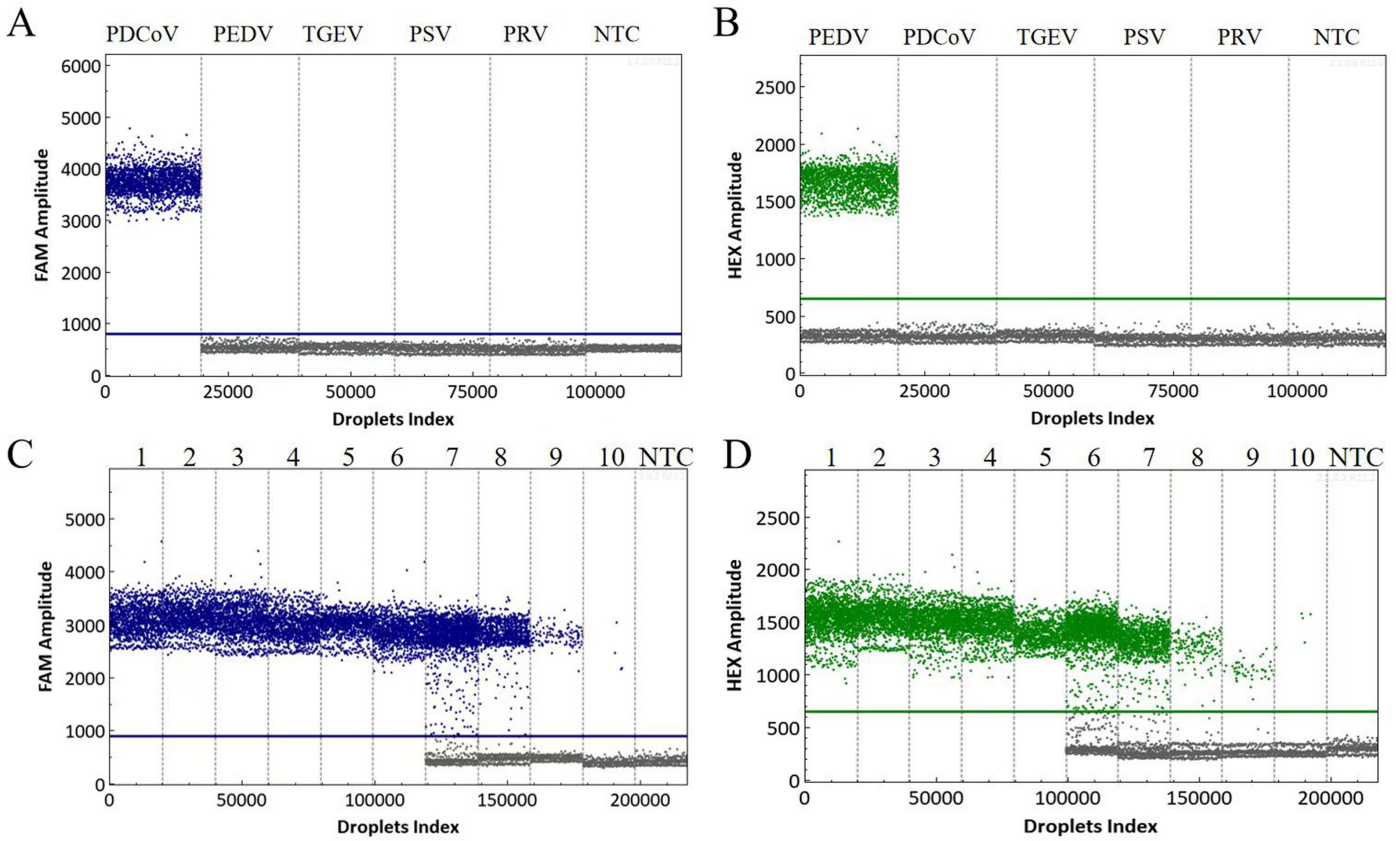
Specificity analysis of the dual cdPCR detection of PDCoV **(A)** and PEDV **(B)**. The fluorescence amplitudes for PDCoV, PEDV, TGEV, PSV, and PRV are shown. Determination of the sensitivity of dual cdPCR detection using serially diluted plasmids: pMD18-T-PDCoV-N (at 1–10:3.36 × 10^9^–3.36 × 10^0^ copies/μL) **(C)**, and pMD18-T-PEDV-M (at 1–10:2.12 × 10^9^–2.12 × 10^0^ copies/μL) **(D)**. NTC: No-template control.

The specificity of the dual cdPCR assay was evaluated using the cDNA/DNA of PDCoV, PEDV, TGEV, PSV, and PRV as templates. RNase-free H_2_O was used as the negative control. The results showed that the established dual cdPCR assay could only detect PDCoV and PEDV, and that the FAM and HEX channel droplets were concentrated while the other viruses were negative (Figures 2A,B). These results demonstrate the detection method’s high level of specificity.

The ten-fold diluted templates of positive standard plasmid pMD18-T-PDCoV-N (at a concentration of 3.36 × 10^9^–3.36 × 10^0^ copies/μL) and pMD18-T-PEDV-M (at a concentration of 2.12 × 10^9^–2.12 × 10^0^ copies/μL) were used for the amplification tests to explore the established dual cdPCR method’s detection limit for PDCoV and PEDV. The detection limit reached the level of a single copy, with an exceptionally high sensitivity (Figures 2C,D), and was more sensitive than the qPCR method (26, 27) established in our laboratory (Supplementary Table S1).

Next, the limit of detection (LoD) for the dual cdPCR assay’s targeting of PDCoV and PEDV was evaluated. The standard plasmids pMD18-T-PDCoV-N and pMD18-T-PEDV-M (at concentrations of 100, 50, 30, 20, 10, 5, 2, 1, 0.5, and 0.1 copies/μL) were subjected to cdPCR and qPCR analysis. As presented in Table 2, the LoD of PDCoV and PEDV (as determined via cdPCR) was approximately 1.83 ± 0.15 copies/μL and 0.99 ± 0.07 copies/μL, respectively, both of which were more sensitive compared to qPCR.

**Table 2:** Detection limits for quantitative real-time PCR (qPCR) and chip digital PCR (cdPCR).

Copies/μL	PDCoV				PEDV			
	qPCR detection		Dual cdPCR detection		qPCR detection		Dual cdPCR detection	
	Detection rate (positive/total)	LoD	Detection rate (positive/ total)	LoD	Detection rate (positive/total)	LoD	Detection rate (positive/ total)	LoD
100	1.00 (12/12)	26.21 ± 2.15	1.00 (12/12)	1.83 ± 0.15	1.00 (12/12)	25.98 ± 1.64	1.00 (12/12)	0.99 ± 0.07
50	1.00 (12/12)		1.00 (12/12)		1.00 (12/12)		1.00 (12/12)	
30	1.00 (12/12)		1.00 (12/12)		1.00 (12/12)		1.00 (12/12)	
20	0.83 (10/12)		1.00 (12/12)		0.92 (11/12)		1.00 (12/12)	
10	0.67 (8/12)		1.00 (12/12)		0.75 (9/12)		1.00 (12/12)	
5	0.33 (4/12)		1.00 (12/12)		0.33 (4/12)		1.00 (12/12)	
2	0.00 (0/12)		1.00 (12/12)		0.08 (1/12)		1.00 (12/12)	
1	0.00 (0/12)		0.75 (9/12)		0.00 (0/12)		1.00 (12/12)	
0.5	0.00 (0/12)		0.25 (3/12)		0.00 (0/12)		0.50 (6/12)	
0.1	0.00 (0/12)		0.00 (0/12)		0.00 (0/12)		0.00 (0/12)	
NTC	0.00 (0/12)		0.00 (0/12)		0.00 (0/12)		0.00 (0/12)	

LoD, the limit of detection; NTC, no-template control.

### 3.3 Linearity and repeatability evaluation of the dual cdPCR assay

**Figure 3:**
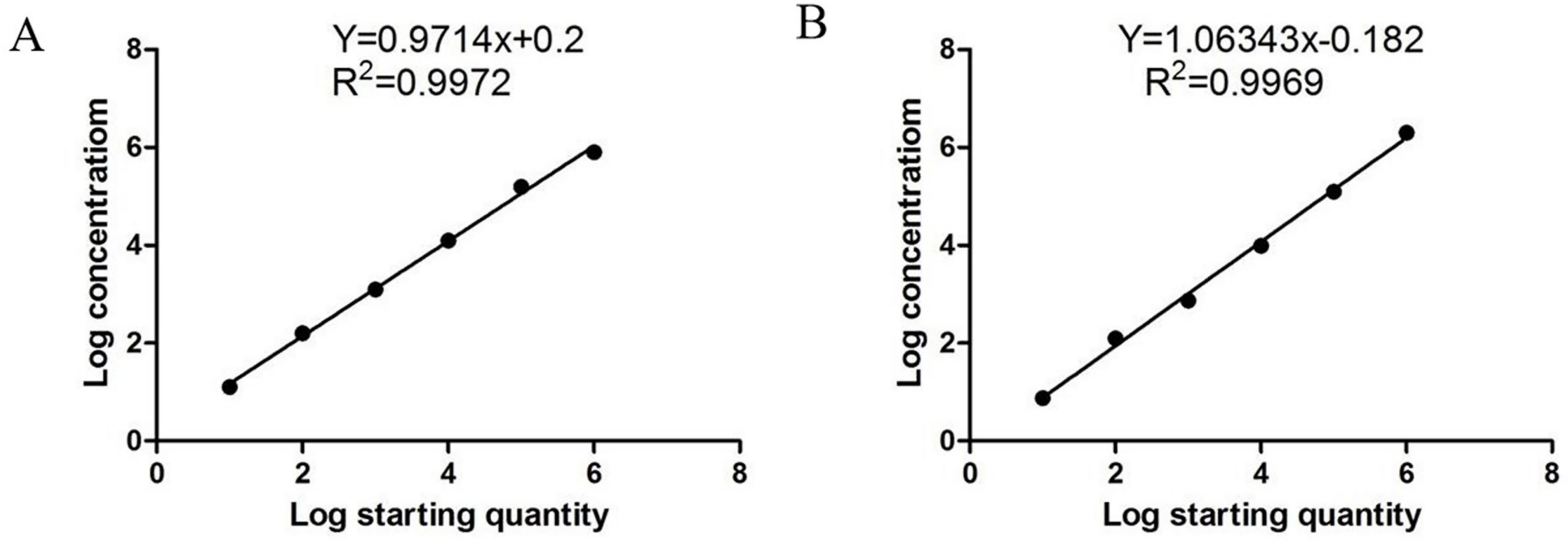
The standard curves of PDCoV **(A)** and PEDV **(B),** drawn from the log absolute concentration against the log starting concentration.

The standard cdPCR curve was constructed using ten-fold diluted PDCoV and PEDV standard plasmids as templates. *R*^2^ was calculated based on the target copy number and measured using a linear relationship for cdPCR. Our results showed that cdPCR had a linear relationship with a PDCoV-*R*^2^ value of 0.9972 and a PEDV-*R*^2^ value of 0.9969 (Figures 3A,B).

The repeatability of the dual cdPCR assay’s results was determined using intra-group and inter-group variability. The coefficient of variation (CV) reflected the outcome of the determination. As shown in Table 3, the intra- and inter-assay CV was less than 6%, indicating that the established cdPCR detection method had a high level of stability.

**Table 3:** Analysis of repeatability and reproducibility of dual cdPCR for PDCoV and PEDV.

Plasmid	Concentration (copies/μL)	Intra-assay of repeatability				Inter-assay of reproducibility			
		Measured values (copies/μL)			CV (%)	Measured values (copies/μL)			CV (%)
PDCoV	3.36 × 10^3^	3,391	3,452	3,504	1.64	3,279	3,367	3,408	1.97
	3.36 × 10^2^	327	344	360	4.80	312	336	349	5.65
	3.36 × 10^1^	35	36	38	4.20	34	35	32	4.54
PEDV	2.12 × 10^3^	2,199	2,203	2088	3.02	2,155	2,284	2056	5.28
	2.12 × 10^2^	224	250	245	5.76	218	215	221	1.38
	2.12 × 10^1^	19	20	21	5.00	22	22	20	5.41

CV, coefficient of variation.

### 3.4 Detection of PDCoV and PEDV in clinical diarrhea samples

A total of 148 clinical diarrhea samples (collected from swine farms in the Henan, Hebei, and Shanxi Provinces) were evaluated. Individual and mixed PDCoV and PEDV infections were analyzed to determine the practicality of the dual cdPCR assay. As shown in Table 4, the cdPCR results indicated that the positive rates were 18.92% (28 out of 148) for PDCoV infection, 23.65% (35 out of 148) for PEDV infection, and 8.11% (12 out of 148) for mixed infections. To eliminate false positives, we retested the four samples that were tested positive by cdPCR but negative by qPCR. The intestinal tissue samples were ground and filtered through a 0.22 μm filter. Next, the filtrate was centrifuged at 30,000 rpm for 120 min at 4 °C. After treatment, all four samples remained cdPCR-positive and qPCR-positive. This discrepancy is most likely attributable to the extremely low viral load in these specimens, which falls below the detection limit of qPCR but remains within the linear range and single-copy sensitivity of cdPCR.

**Table 4:** Detection results for clinical diarrhea samples.

*N* = 148	Dual cdPCR detection		qPCR detection	
	Positive	Positive rate (%)	Positive	Positive rate (%)
PDCoV	28	18.92	25	16.89
PEDV	35	23.65	33	22.30
PDCoV+PEDV	12	8.11	11	7.43

### 3.5 cdPCR detection of PDCoV and PEDV aerosols

**Figure 4:**
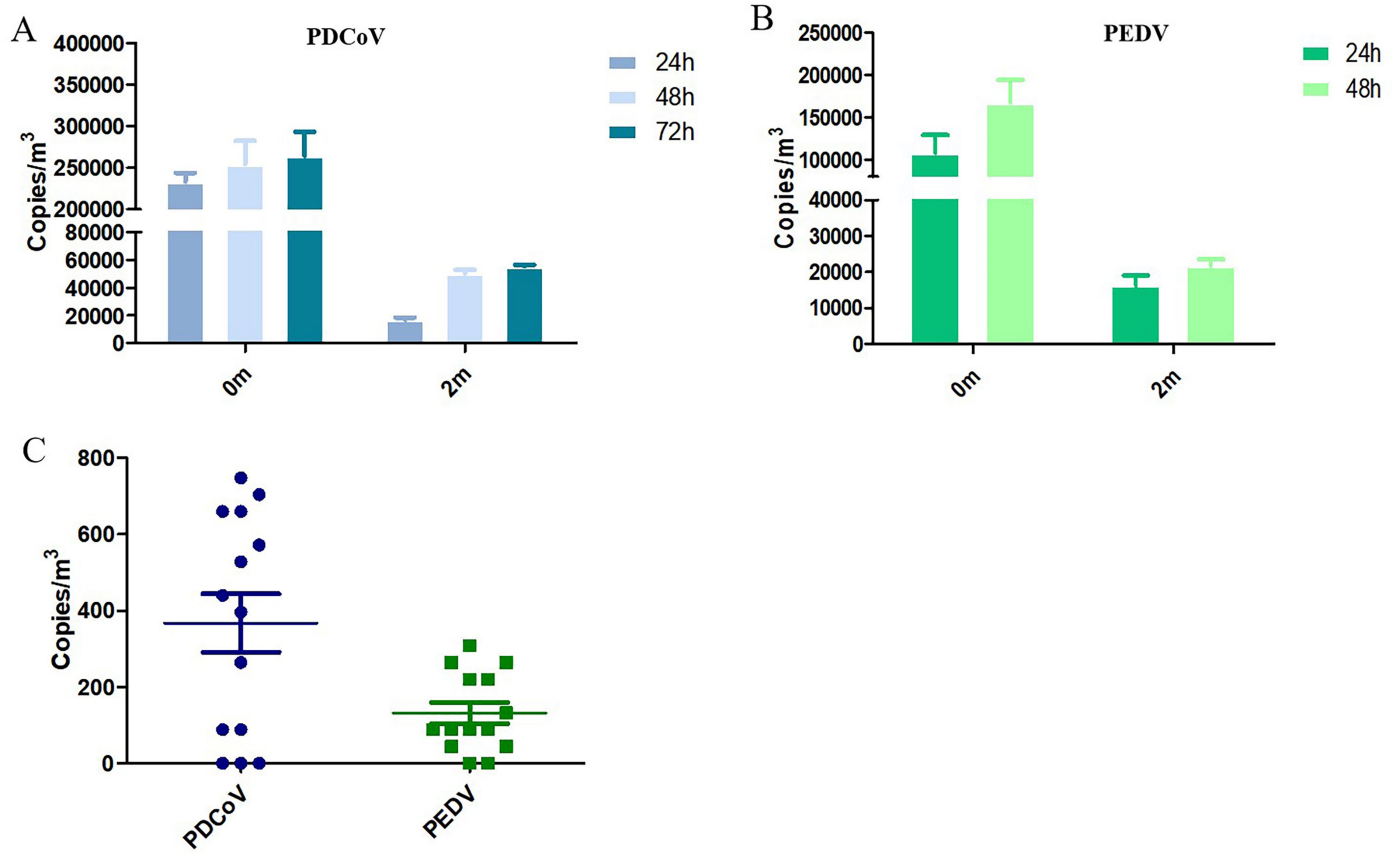
PDCoV and PEDV cdPCR results from air samples collected from experimentally infected piglets and manure trench. **(A,B)** Total PDCoV and PEDV virions in the aerosol results from air samples collected from experimentally infected piglets. Results are reported as estimated PDCoV and PEDV RNA copies per m3 of air and hours post-inoculation. In the PEDV-challenge group, piglets died within 48–72 h, and only 24 h and 48 h were monitored. **(C)** Results from PDCoV and PEDV cdPCR positive air samples collected from the area above the manure trench. Error bars represent the standard error of the mean (SEM).

In farming environments, pathogenic microbial aerosols can cause environmental pollution and infectious diseases (28). Recent studies have highlighted the potential airborne transmissibility of PDCoV (13). Researchers have emphasized the hazards of airborne PEDV transmission and have confirmed its underlying mechanisms (12). The efficient sampling and accurate detection of microorganisms in aerosols are essential for analyzing the properties of aerosols and assessing their hazards. This study utilized a wetted wall cyclone aerosol sampler and a dual cdPCR method for detecting PDCoV and PEDV aerosols. At 24 h post-infection, the piglets exhibited symptoms such as vomiting and diarrhea. In the challenge group with a horizontal distance of 0 meters, the estimated number of RNA copies of PDCoV and PEDV per m^3^ reached 10^5^ (Figures 4A,B). At further distances from the viral source, the amount of virion and genetic material in the air decreased significantly. The negative control samples tested negative. These results establish a strong basis for investigating the airborne transmission of PDCoV and PEDV.

Studies have confirmed that PEDV can survive for up to 9 months in fecal matter. Bio-aerosolized virus particles released from infected manure may contaminate the surrounding environment (29). In our study, we monitored a pig farm that had been vacant for 6 months due to a previous PEDV and PDCoV outbreak. We applied a dual cdPCR method to analyze air samples collected from the area above the manure trench. As shown in Figure 4C, low concentrations of PDCoV and PEDV nucleic acids were detected in these air samples. Furthermore, we also conducted the distribution of PDCoV and PEDV in the swine manure treatment system (Supplementary Figure S1).

## 4 Discussion

PEDV and PDCoV are members of the *alphacoronavirus* and *deltacoronavirus* genera, respectively, both belonging to the *Coronaviridae* family (30, 31). Both viruses can cause lethal watery diarrhea in neonatal pigs (32). Singular infection and coinfection with PDCoV and PEDV are ubiquitous in pig farms, causing massive economic losses to the swine industry worldwide (33). What’s worse, PDCoV has been detected and isolated in plasma samples taken from three Haitian children with acute febrile illness, thus posing a potential threat to public health (34). It is therefore imperative to continuously monitor PDCoV and PEDV and their epidemic variants to inform effective control strategies. Numerous etiological and serological techniques for identifying PDCoV and PEDV have been established. Although these approaches have significantly contributed to the accurate detection of PDCoV and PEDV, developing more sensitive and reliable detection methods would further enhance the swine industry’s ability to circumvent outbreaks.

As a third-generation PCR detection technology, digital PCR is an accurate, quantitative, single-copy detection method (35, 36). Digital PCR does not require standard curves or internal reference genes, and it has a better tolerance to PCR inhibitors (17, 37). Digital PCR is used extensively across all aspects of biology, including malignancy surveillance, pathogen diagnosis, nucleic acid quantification, vaccine evaluation, and mutant gene identification (38–40). Shi et al. (24) developed a multiplex crystal digital PCR method for the simultaneous detection of African swine fever virus (ASFV), classical swine fever virus (CSFV), and porcine respiratory syndrome virus (PRRSV). Dioni et al. (41) developed a chip digital PCR method to quantify SARS-CoV-2 at low viral loads. A single digital PCR method has been established for detecting PDCoV and PEDV (42, 43). However, a multiplex digital PCR method for the differential detection of these two pathogens has not yet been developed. Therefore, we developed a novel dual-channel microfluidic cdPCR method using FAM and HEX fluorescent dyes to detect PDCoV and PEDV effectively and accurately.

Firstly, we optimized the concentrations of primers and probes, as well as the annealing temperature. We then evaluated the assay’s specificity, sensitivity, and repeatability. The experimental results indicate that this method could specifically detect PDCoV and PEDV, but could not detect the nucleic acids of TGEV, PSV, and PRV. The results of specificity analysis are consistent with those obtained using methods established by other researchers (26, 27) (Supplementary Table S2). The assay’s sensitivity for PDCoV and PEDV was much higher than that of qPCR, with an LoD of 1.83 ± 0.15 copies/μL and 0.99 ± 0.07 copies/μL, respectively. This cdPCR assay presents a significant advantage in detecting low quantities of templates compared to qPCR (44). In this study, all four clinical diarrheal samples tested negative via qPCR but positive via dual-probe cdPCR. However, after sample filtration and concentration increased viral load while removing inhibitory substances, qPCR detection yielded positive results. This demonstrates cdPCR’s superior sensitivity compared to qPCR, achieved through partitioning samples into reaction chambers each containing ≤1 copy of the target sequence. This approach inherently mitigates interference from complex matrices (e.g., bile salt- and polysaccharide-rich intestinal samples) while maintaining quantitation accuracy (45). Compared to qPCR, cdPCR remains relatively expensive. However, since it is suitable for early virus detection, its implementation can reduce prevention and control costs. With the development of duplex and multiplex cdPCR and reduced cost per sample, this method is being applied more frequently in laboratory settings (46).

PDCoV and PEDV have the potential for aerosol transmission in addition to fecal-oral transmission (12, 13). The transmission of viral aerosols can lead to widespread disease epidemics (47, 48). Therefore, sensitive and accurate methods for detecting PDCoV and PEDV aerosols are essential for preventing widespread epidemics of these two viruses. Digital PCR methods with high levels of sensitivity and accuracy are commonly used to detect coronavirus aerosols. Liu et al. (49) and Kim et al. (50) developed digital PCR detection methods for the precise detection of SARS-CoV-2 aerosols based on the SARS-CoV-2 ORF1a/b and N genes. In this study, we used an established cdPCR method to detect PDCoV and PEDV in air samples around infected piglets (at horizontal distances of 0 and 2 meters). At 24 h post-inoculation, the PDCoV and PEDV RNA copies per m^3^ of air above the infected group (at a horizontal distance of 0 meters) reached 10^5^. The virus particle content decreased tenfold at a distance of 2 meters. This indicates that the virus particle content in air decreases sharply at increasing distances from the viral source. Although a few studies have investigated the airborne transmission patterns of porcine enteric coronaviruses, more studies have been done on the airborne transmission patterns of human coronaviruses. Teleman et al. (51) and Wessel et al. (52) found that the critical distance of viral droplets is 1 to 2 meters away from the infection source. Within that critical distance, the virus content decreases at further distances from the viral source—which aligns with our results. Our cdPCR method provides a foundation for studying the airborne transmission patterns of PDCoV and PEDV.

## 5 Conclusion

We established a novel dual cdPCR method for the simultaneous detection of PDCoV and PEDV. This cdPCR method demonstrates adequate degrees of sensitivity, specificity, stability, and accuracy. It has the potential to serve as a rapid and reliable tool for the early detection of PDCoV and PEDV infections. Moreover, it provides a foundation for the further investigation of these viruses’ airborne transmission patterns, as well as technical support for the prevention and control of diseases caused by PDCoV and PEDV.

## Data Availability

The original contributions presented in the study are included in the article/Supplementary material, further inquiries can be directed to the corresponding authors.
