# Supplementary material for: Establishment and application of a dual chip digital PCR assay for detection of PDCoV and PEDV
Source: Front Vet Sci. 2025 Sep 15;12:1655079. doi: 10.3389/fvets.2025.1655079 (PMC12477013; doi:10.3389/fvets.2025.1655079)
Supplement: Supplementary file 1 [file Table_1.doc]

**Supplemental Information for:**

**Establishment and application of a dual chip** **digital PCR Assay for detection of PDCoV and PEDV**

Yue Zhang^1,4†^, Fangting Dong^1†^, Yuhang Zhang^1^, Yutong Feng^1^, Jinwang Hu^1^, Yuhang Li^1^, Lu Xia^1,3^, Shaopo Zu^1,3^, Hao Lu^2*^ and Zhanyong Wei^1,4*^

† Yue Zhang and Fangting Dong contributed equally to this work.

* Correspondence: luhao79@outlook.com; weizhanyong@henau.edu.cn

^1^ College of Veterinary Medicine, Henan Agricultural University, Zhengzhou, China,

^2^ Molecule Biology Laboratory of Zhengzhou Normal University, Zhengzhou, China,

^3^ Henan Province Key Laboratory of Animal Food Pathogens Surveillance, Zhengzhou, China,

^4^ Ministry of Education Key Laboratory for Animal Pathogens and Biosafety, Zhengzhou, China

**TABLE S1. Sensitivity comparison of the qPCR and cdPCR detection methods.**

| **cDNA** | **Concentration (copies/μL)** | **Measured values^a^** | |
| --- | --- | --- | --- |
|  |  | **qPCR detection**  **mean (Ct) ± SD** | **Dual cdPCR detection**  **mean (copies μL^-1^) ± SD** |
| PDCoV | 3.36 × 10^3^ | 26.85 ± 0.09 | 3370.67 ± 85.96 |
|  | 3.36 × 10^2^ | 31.75 ± 0.31 | 342.17 ± 8.60 |
|  | 3.36 × 10^1^ | 37.17 ± 1.84 | 36.66 ± 1.98 |
|  | 3.36 × 10^0^ | NA | 4.18 ± 0.54 |
| PEDV | 2.12 × 10^3^ | 26.85 ± 0.10 | 2105.43 ± 63.30 |
|  | 2.12 × 10^2^ | 30.56 ± 0.07 | 222.71 ± 4.19 |
|  | 2.12 × 10^1^ | 34.70 ± 0.54 | 22.15 ± 0.24 |
|  | 2.12 × 10^0^ | NA | 3.18 ± 0.83 |

**^a^** Compare the cd-PCR detection method with previously reported q-PCR methods for PDCoV^[1]^ and PEDV^[2]^ .

"NA" represents “No Amplification”.

**TABLE S2. Specificity comparison of the qPCR and cdPCR detection methods.**

| **cDNA** | **Measured valuesa** | | | |
| --- | --- | --- | --- | --- |
|  | **qPCR detection**  **mean (Ct) ± SD** | | **Dual cdPCR detection**  **mean (copies μL-1) ± SD** | |
| PDCoV | 30.14 ± 0.46 | NA | 617.18 ± 9.50 | NA |
| PEDV | NA | 29.42 ± 0.12 | NA | 378.32 ± 10.26 |
| TGEV | NA | NA | NA | NA |
| PSV | NA | NA | NA | NA |
| PRV | NA | NA | NA | NA |

**^a^** Compare the cd-PCR detection method with previously reported q-PCR methods for PDCoV^[1]^ and PEDV^[2]^ .

"NA" represents “No Amplification”.

**Reference:**

[1] Lu S J, Ma M Y, Yan X G, et al. Development and application of a low-priced duplex quantitative PCR assay based on SYBR Green I for the simultaneous detection of porcine deltacoronavirus and porcine sapelovirus[J]. Vet Med (Praha), 2023, 68(3): 106–15.

[2] Zheng L L, Cui J T, Han H Y, et al. Development of a duplex SYBR GreenⅠ based real-time PCR assay for detection of porcine epidemic diarrhea virus and porcine bocavirus3/4/5[J]. Mol Cell Probes, 2020, 51: 101544.

**
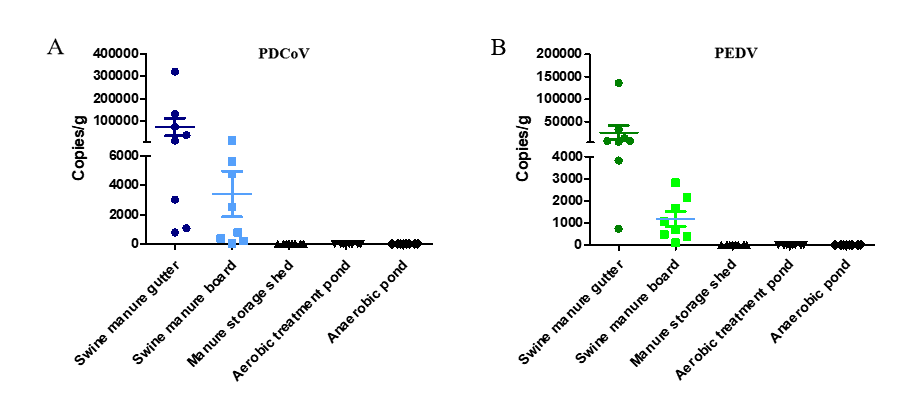
**

**FIGURE S1.** **The distribution of PDCoV and PEDV** **in the swine manure treatment system.**

In our study, we monitored a pig farm that had been vacant for six months due to a previous PDCoV and PEDV outbreak, and then analyzed the distribution of these viruses in the swine manure treatment system. The results showed that PDCoV and PEDV were detected in the manure gutter and manure board with 100% detection rates, but were absent from the manure treatment areas, including the manure storage shed, aerobic treatment pond, and anaerobic pond. Generally, the persistence of viral nucleic acids is directly influenced by environmental factors. The manure gutters of pig pens are cold, damp, and poorly ventilated; these conditions hinder viral nucleic acid degradation. In contrast, the manure storage shed, aerobic treatment pond, and anaerobic pond, all of which undergo long-term heat treatment and fermentation, showed no viral nucleic acids detected. Error bars represent the standard error of the mean (SEM).
